# The Prevention of Milk Adulteration

**Published:** 1907-06-01

**Authors:** 


### MEDICO-LEGAL DEPARTMENT.

### The Prevention of Milk Adulteration.

### MEDICAL OFFICERS AND OTHER LEGAL POWERS.

Section 9 of the Sale of Food and Drugs Act, 1875, enacts that no person shall, with the intent that the same may be sold in its altered state without notice, abstract from an article of food any part of it so as to affect injuriously its quality, substance, or nature, and no person shall sell any article so altered without making disclosure of the alteration, under a penalty in each case not exceeding £20.

The Board of Agriculture, under Section 4 of the Sale of Food and Drugs Act, 1899, issued in 1901 certain regulations with respect to the sale of milk. These regulations provide that where a sample of milk (not being milk sold as skimmed or separated, or condensed milk) contains less than 3 per cent. of milk fat, it shall be presumed for the purposes of the Sale of Food and Drugs Acts, 1875 to 1899, until the contrary is proved, that the milk is not genuine by reason of the abstraction therefrom of milk fat, or the addition thereto of water. Where a sample of milk (not being milk sold as skimmed, or separated, or condensed milk) contains less than 8.5 per cent. of milk solids other than milk fat, it shall be presumed, for the purposes of the above Acts, until the contrary is proved, that the milk is not genuine by reason of the abstraction therefrom of milk solids, other than milk fat, or the addition thereto of water. Where a sample of skimmed or separated milk (not being condensed milk) contains less than 9 per cent. of milk solids, it shall be presumed for the purposes of the above Acts, until the contrary is proved, that the milk is not genuine by reason of the abstraction therefrom of milk solids other than milk fat, or the addition thereto of water. Presumptions are, of course, capable of being rebutted. Milk below the standard is not necessarily adulterated, and milk above the standard is not necessarily genuine.

Although the quality of genuine milk offered for sale will usually be well above the official limits of milk fat and non-fatty solids, there may occasionally, and especially in certain seasons of the year, be cases in which a sample of genuine milk may fall below those limits. To meet cases of this kind the Board of Agriculture suggest that in the absence of any special circumstances indicating that the case is a fraudulent one, the local authority might, in the first instance, call the vendor's attention to the analyst's report, and ask him whether he desires to offer any explanation, and if the explanation is one they are able to accept they might, in the exercise of their discretion, refrain from the institution of proceedings or withdraw any summons which, in order to prevent the failure of proceedings, by reason of the time limit imposed by the Act, it may have been necessary to take out. But it may be desirable that further samples of milk should be taken in such cases, in order that a satisfactory conclusion as to the character of the milk supplied may be arrived at.

The Milk Regulations Committee reported that the evidence submitted to them went to show that it was a common practice to add gelatin to cream for the purpose of giving it a fictitious appearance of richness or thickness. Local authorities are urged to take steps to ascertain whether this form of adulteration is practised within their districts, and if a public analyst reports the presence of gelatin or other similar substances in a sample of cream, the local authority concerned should consider whether the case is one in which proceedings might not with advantage be instituted under Section 6 of the Sale of Food and Drugs Act, 1875.

Milk is an article which varies very considerably in composition, the differences arising partly from the breed of the cow, and partly from the way in which the animal is fed and housed. There is also a perceptible difference between the milk given by the same cow at different times of the year. Separated milk is sometimes sold as skimmed milk, and such a sale is a fraud upon the purchaser, because considerably more fat can be removed by a separator than by the ordinary method of skimming, and is punishable under the Acts.

If at the time of sale the vendor gives notice to the purchaser that the cream has been abstracted, he is not liable under Section 6. But if no such notice has been given it is no defence to a prosecution under Section 6 for him to plead that he had no knowledge of the alteration at the time of sale—unless, of course, he can produce a written warranty in accordance with the provisions of Section 25 of the Sale of Food and Drugs Act, 1875. It was held in *Dyke* v. *Gower* (1892, 1 Q.B. 220) that the words "so altered" in Section 9 refer to a physical alteration of the article, irrespective of the intent with which the alteration is made. In *Morris* v. *Corbett* (56 J. P., 649) the servant of C., a dairyman, being short in his supply of milk, bought two gallons from another dairyman and mixed it with his own and sold the same to customers. It was held that though neither C. nor C.'s servant knew, or had reason to suspect the milk was adulterated, this was no defence.

A special contract between vendor and purchaser as to the quality of the articles to be delivered by the former is no bar to a conviction under Section 9 of the Act of 1875. In *Fecitt* v. *Walsh* (1891, 2 Q.B. 304) the appellant contracted to supply milk to a workhouse at a certain price; by the contract the milk was to contain a certain percentage of cream; it was to be tested on each delivery, and a reduction was to be made in the price in respect of any deficiency in cream. While the daily supply, contained in five cans, was being delivered at workhouses the respondent procured a sample from each of the five cans; there being a large deficiency of cream in two of the samples the respondent subsequently laid two separate informations against the appellant in respect of these two samples. The justices convicted the appellant in a separate penalty upon each information. It was held that the procuring of each sample was a separate transaction, that the appellant had committed a separate offence as to each case in respect of which an information was laid. That the separate informations were therefore properly laid, and the convictions were right. In *Jones* v. *Davies* (69 L.T. 497) the respondent sold to the appellant a tin of skimmed milk, which purported to be, and was sold as marked upon a label upon the tin, "Condensed milk." On another part of the label in smaller print it stated that "this tin contains skimmed milk." As to whether there had been a sufficient disclosure within the meaning of Section 9 it was held that the label on the tin was a disclosure of the contents of the tins. In *Platt* v. *Tyler* (58 J.P. 71) P. was charged with selling condensed milk, from which 80 per cent. of fat had been abstracted without making disclosures of the alteration. The purchaser was told it was skimmed milk and pointed to a label on the tin, which, in smaller type, stated the milk to be skimmed. The justices held the disclosure insufficient, and convicted P. It was held that the justices were wrong, and that the disclosure was sufficient.

In *Spiers & Pond* v. *Bennett* (1896, 2 Q.B. 65) milk was delivered under contract at a refreshment room of the appellants; it was handed in a can to one of their servants, who poured a portion of it into a churn, which was placed on the counter for the purpose of the milk being sold to customers; it was so poured that a less proportion of cream was in the milk that went into the churn than in the milk which remained in the can. The respondent bought a glass of milk at the counter; the milk was drawn from the churn, and on analysis showed a deficiency of 17 per cent. of cream. Upon the glass in which the milk was served were engraved the words: "Not guaranteed as new or pure milk or with all its cream; see notices"; and upon the refreshment counter was a printed notice to the effect that all milk sold by the appellant was purchased by them under a warranty of its purity and genuine quality; that they took all possible precautions to ensure its supply to their customers in proper condition, but were unable to guarantee it as new, pure, or with all its cream, and did not, therefore, sell it as such. It was held that, assuming that the facts showed an abstraction from the milk, there had been a sufficient disclosure by the appellants of the alteration to satisfy the requirements of the section.

